# History of Nephrotic Syndrome and Evolution of its Treatment

**DOI:** 10.3389/fped.2016.00056

**Published:** 2016-05-30

**Authors:** Abhijeet Pal, Frederick Kaskel

**Affiliations:** ^1^Division of Pediatric Nephrology, Children’s Hospital at Montefiore, Albert Einstein College of Medicine, New York, NY, USA

**Keywords:** nephrotic syndrome, early recognition, evaluation, natural history, treatments

## Abstract

The recognition, evaluation, and early treatment of nephrotic syndrome in infants and children originate from physicians dating back to Hippocrates. It took nearly another 1000 years before the condition was described for its massive edema requiring treatment with herbs and other remedies. A rich history of observations and interpretations followed over the course of centuries until the recognition of the combination of clinical findings of foamy urine and swelling of the body, and measurements of urinary protein and blood analyses showed the phenotypic characteristics of the syndrome that were eventually linked to the early anatomic descriptions from first kidney autopsies and then renal biopsy analyses. Coincident with these findings were a series of treatment modalities involving the use of natural compounds to a host of immunosuppressive agents that are applied today. With the advent of molecular and precision medicine, the field is poised to make major advances in our understanding and effective treatment of nephrotic syndrome and prevent its long-term sequelae.

## History of Nephrotic Syndrome

Nephrotic syndrome, as we know it today, is a combination of proteinuria, hypoalbuminemia, hyperlipidemia, and edema, a concept that took some time to be developed. Interestingly, the effective treatments became available only recently in the mid 1900s, with the advent of steroids, antibiotics, diuretics, and other immunomodulators. Even today, there is a gap in our understanding of the etiology(s) of nephrotic syndrome of childhood, and better treatments are still required in the more resistant forms. Nephrotic syndrome as such is a combination of clinical and laboratories findings that is seen with a variety of pathologic lesions affecting the glomerulus.

Generalized edema, referred to as dropsy in the earlier literature, has been documented from the times of Hippocrates (1, 2), although the differentiation between various causes (heart vs. liver vs. nutritional disturbances vs. renal abnormalities) was not made. One observation by Hippocrates was: “when bubbles settle on the surface of the urine, it indicates a disease of the kidney and that the disease will be protracted” (1). Cornelus Roelans of Belgium described in 1484 a child with nephrotic syndrome and “whole body swelling.” He went on to recommend the treatment as follows: “take the tops of elder plant and daneswort, cook in white wine and wrap the child in hot clothes by applying the poultice in whole or in part, and so cure him” (3).

During the sixteenth to eighteenth centuries, dropsy was considered a disease *per se*, the patient had dropsy, without differentiating between the causes (4). One of the first accurate descriptions of nephrotic syndrome in children was made by Theodore Zwinger of Basel in 1722 (5). He also noted decreased urine output and attributed this to “obstruction and compression of the tubules of the kidney,” thus placing the seat of the disease in the kidney, since at that time, it was recognized that in pediatric practice heart and liver disease rarely manifest. His important observation had little influence on the scientific community, and his findings were not quoted by any author in the following 200 years. Of note, these observations were made even before Morgagni was able to establish the idea that disease might arise from specific organs (6). Morgagni’s disciple William Heberden went on to say: “Dropsy is very rarely an original distemper but generally a symptom of some other which is too often incurable” (7).

Later in the eighteenth century, dropsy was divided into those dependent on morbid viscera (liver and heart) and a general form, supposedly inflammatory. With humoralism, a prevalent concept in Europe at that time, bloodletting was a common practice for these so called “inflammatory diseases” and was a commonly prescribed. Around the same time, several observers [namely, Cotugno (8), Cruikshank (9), Wells (10), and Brande (9)] noted coagulability of urine in the patients. It was finally in 1827 that Richard Bright (1789–1858) was able to put together the triad of generalized edema, proteinuria, and kidney disease, as presenting features of this disease (11). John Bostock, a colleague of Bright, also noted that when protein in the urine was highest, it was lowest in the serum (11). Christison confirmed these findings in 1829 (12). Hence by 1830, nephrotic syndrome of profound albuminuria, hypoalbuminemia, and edema, resulting from diseased kidneys was established. Subsequently, on the postmortem analysis, these kidneys revealed a diseased state. Bright described three varieties of postmortem appearance of the kidneys, Chritison seven, Pierre Rayer six in 1840, and Carl Rokitansky no less than eight in 1846, including the “specknierre” or bacon kidney, recognized later as amyloidosis (9).

Over the next few years, the various lipid abnormalities in nephrotic syndrome became the forefront of discussion. Latescent or milky appearance of the serum was known to be present in nephrotic syndrome (noted with bloodletting sessions). Christison showed this to be from ether soluble fat (13). In 1846, Johnson not only described the fatty nature of the kidneys on gross appearance in many such patients but also fat globules and fat casts in the tubules (14). More attention was given to the cellular and tubular components of the kidneys as they were more obvious by the histological techniques available at that time.

Virchow introduced the term “parenchymatous nephritis” for a pathological picture with primary tubular involvement (15). With advances in microscopy, glomerular involvement became clearer along with that of the parenchyma. In acute nephritis, the presence of pale exsanguinated glomeruli was long known. In 1872, Klebs coined the term “glomerulonephritis” to describe the exudative glomerular changes seen under the microscope (16). In 1905, the term “nephrosis” was coined by Müller to describe all “non-inflammatory” diseases of the kidney as a substitute for parenchymatous nephritis, contrasting it with exudative and inflammatory disease, which would retain the name nephritis (17). This concept of “nephritis in contrast to nephrosis” was further popularized by F. Volhard, T. Fahr, and C. Munk.

## Treatments for Nephrotic Syndrome

Introduction of the renal biopsy in the mainstream clinical practice of nephrology during the 1950s–1960s added a new dimension to the understanding of the histological findings in nephrotic syndrome. The histological classifications were based on the light microscopic appearance and included membranous glomerulonephritis, proliferative glomerulonephritis, a mixed membranous and proliferative glomerulonephritis, diabetic nephropathy with hyaline nodules, and focal segmental glomerular sclerosis; these are summarized in a case series from 1958 (18). The availability of electron microscopy and immunofluorescent localization of proteins was a welcomed coincidence. These new techniques transformed the ideas on morphology and pathogenesis, and made definite correlations between clinical presentation and pathological picture. The 1961, a Ciba Foundation symposium on the use of the renal biopsy was a landmark in these regards (19).

Antibiotics played an important role in treating the infections that arose from nephrotic syndrome, and the mortality rate reduced drastically from two-third to 35% (20). It is important to point out that prior to availability of steroids multiple desperate treatments were tried. A study from Boston noted the various treatments that were attempted from 1926 to 1948 for nephrotic syndrome. Dietary modification and low salt diet were probably the most effective treatments at that time. There were some weak mercurial diuretics with little if any action. Other drastic measures, such as the induction of measles and vaccinia, were instituted. Many of the children inoculated had some form of post-infection diuresis and decrease in proteinuria (11 out of 14), only to recur. Other supportive measure included blood transfusions, antibiotic therapy, treatment with thyroid extract, decapsulation of the kidney, testosterone, multiple vitamins, horse antiserum, and parathyroid hormone.

Steroid hormones were first isolated and identified in 1936 (21, 22). By 1946, cortisone was first prepared by partial synthesis from bile acids (23). On the other hand, adrenocorticotropic hormone (ACTH) was extracted from pig and sheep pituitary gland by isoelectric precipitation (22, 24) in the 1940s. One preparation of ACTH was commercially available as sterile powder reconstituted with isotonic saline for intramuscular injection. In the early 1950s, intramuscular injections of Cortisone (25) and ACTH (26) were first being used for treatment for nephrotic syndrome in children by Arneil and Wilson from Glasgow. These were relatively short courses, 5 days of daily 100–300 mg of intramuscular cortisone in the first study and 40–80 mg of an ACTH intramuscular injection for 12 days in the second study (25, 26). Later on, prednisone was first synthesized by oxidation of cortisone (27, 28). ACTH and cortisone were quickly replaced by prednisolone and prednisone as they could be administered orally without the need for daily injections (29, 30). With the advent of steroid therapy, mortality from nephrotic syndrome dramatically decreased to 3% (31).

An important series of studies, in the era after renal biopsy and steroids, to better understand the management of childhood nephrotic syndrome were heralded by the International Study of Kidney Disease in Children (ISKDC) established in 1965 with participants from North America, Europe, and Asia. A series of prospective, multicenter cooperative studies by the ISKDC established definitions, clinicopathological correlations, and recommendations for therapy that provided a basis for diagnosis and management of pediatric nephrotic syndrome that persists today. Between January 1967 and June 1974, children with the nephrotic syndrome who were older than 12 weeks and younger than 16 years were enrolled into the clinical survey from the 24 participating clinics (32–34). Of the 521 entered, 76.6% had minimal-change nephrotic syndrome, 7.5% had membranoproliferative glomerulonephritis, and 6.9% had focal segmental glomerulosclerosis (35). All participants had biopsies prior to starting steroid therapy with prednisone. Initial treatment was 60 mg/24 h/m^2^ (maximum dosage 80 mg/24 h) in divided doses for 4 weeks, followed by 40 mg/24 h/m^2^ in divided doses, three consecutive days out of seven for 4 weeks. A seminal observation was made that patients with non-minimal lesions had a varied and limited response to steroids (36).

Azathioprine, a purine synthesis inhibitor, was also tested by this group in a randomized placebo-controlled trial (37). Patients that were considered to be early non-responders to steroids (not responding to the initial 8-week therapy) or frequently relapsing were randomized to receive every other day prednisone with azathioprine (test group) or placebo (control group). No significant decrease in proteinuria or number of relapses was noted in the test group vs. the control group (37).

The use of an alkylating agent in steroid-resistant nephrotic syndrome was first initiated with nitrogen mustard as early as 1958 (38). This was extended to other alkylating agents, cyclophosphamide and chlorambucil, in the 1960s and prompted the ISKDC to conduct a randomized control trial to define the role of cyclophosphamide in children with early non-responders and frequently relapsing nephrotic syndrome. Cyclophosphamide was shown to remit proteinuria in early non-responders and decrease the number of relapses of nephrotic syndrome (39). Thus, it proved to be an important agent to decrease steroid use and prevent steroid toxicity, although it caused gonadal failure in post-pubertal males (39). Chorambucil, although noted to be as effective as cyclophosphamide (40), had concerning side effects of acute leukemia and renal carcinoma (41).

The initial 8-week steroid regimens (4 weeks daily and 4 weeks every other day) were then compared with a longer steroid treatment duration in the late 1980s (42). Mounting evidence supported a longer duration of steroids, which helped decrease the number of future relapses and steroid dependence, and the initial 8-week steroid regimen has been recommended to be increased to at least 12 weeks (43, 44).

Levamisole, known for its anthelmintic, was first reported to be used in children with nephrotic syndrome in 1980 (45) and continues to be used as a steroid sparing agent in many countries. Cyclosporine A (CsA), a calcineurin inhibitor, was first isolated from the fungus *Tolypocladium inflatum* found in a soil sample obtained in 1969 from Norway, by Hans Peter Frey (46). It was initially used in humans for renal transplantation in 1978 and has since changed the face of transplantation (47). The first use for CsA was reported in 1986 among adults with difficult to treat nephrotic syndrome (48). By the late 1980s, there were reports of its successful use in children with steroid-resistant or steroid-dependent nephrotic syndrome that had not responded well to alkylating agents with the target levels of CsA between 50 and 200 ng/ml (49, 50).

Tacrolimus, another calcineurin inhibitor, was first extracted from *Streptomyces tsukubaenis* in 1987 by a Japanese group (51) and was initially used as a drug in human organ transplantation. Its use for nephrotic syndrome was first started in adults in the early 1990s (52–54) and then reported to be used children by the early 2000s (55–57). It was shown to be similar to CsA with regard to efficacy and renal toxicity but without the cosmetic side effects of hirsutism and gingival hypertrophy (58).

Mycophenolate mofetil (cellcept^®^) and, more recently, mycophenolate sodium (myfortic^®^) are prodrug forms of mycophenolic acid, another purine synthesis inhibitor, identified from *Penicillium* species. It was first reported to be used in pediatric nephrotic syndrome in 2000 (59) after being successfully applied in other glomerular diseases (60) and renal transplantation. It has been shown to be useful in steroid-dependent and frequently relapsing steroid-sensitive nephrotic syndrome, as a first-line agent, although there is evidence to indicate that it is probably less effective that calcineurin inhibitors (43, 61–63).

Rituximab, a monoclonal antibody against a B-lymphocyte antigen CD20, came into the armamentarium after case reports describing the incidental finding of a beneficial effect of rituximab on childhood nephrotic syndrome (64). Benz et al. first used rituximab to treat ITP, resistant to steroids and immunoglobulin therapy, in a 16-year-old boy who also suffered from steroid-dependent nephrotic syndrome. As well as effectively resolving the ITP, rituximab also improved the nephrotic syndrome, inducing a relapse-free period for over 12 months on low-dose CsA (65). In the other two cases, rituximab was used to treat post-transplant lymphoproliferative disease (PTLD) in boys who also had recurrence of FSGS in their transplants (66, 67), with beneficial effects on proteinuria. Its efficacy was recently demonstrated as a steroid-sparing agent in childhood onset frequently relapsing and steroid-dependent nephrotic syndrome by a multicenter, double-blind, randomized, and placebo-controlled trial (68) conducted by Iijima et al. Patients in the rituximab-treated group were relapse-free for prolonged period of time despite being weaned off other immunosuppressants such as mycophenolate or cyclosporine. There have been few case reports of its efficacy in refractory primary FSGS; however, controlled clinical trails are needed to define its exact role (69, 70). It has been proven to be effective in combination with plasmapheresis for post-transplant recurrence of FSGS (70, 71). Part of this effect may be mediated by its direct action on the podocyte (72).

## Future Treatments and Conclusion

Interestingly, ACTH is currently remerging as a potential treatment for nephrotic syndrome. In Europe, it is available as a synthetic depot formulation (Synacthen^®^) and in the US as a highly purified formulation (Acthar^®^ gel) from porcine or bovine sources (73). There are reports on its efficacy in multiple causes of nephrotic syndrome, including idiopathic membranous nephropathy, FSGS, minimal-change disease, and mesangial glomerulonephritis, with a response rate varying from 29 to 100% (73–79). Recently, it has been shown to be particularly beneficial in idiopathic membranous nephropathy. Although the exact mechanism of action for ACTH is not known, it is thought to act directly on the podocytes *via* melanocortin receptors on the podocytes. There is need for a multicenter randomized control trial to assess its use in idiopathic nephrotic syndrome.

With discovery of various gene defects associated with nephrotic syndrome, there is an expanding knowledge of various podocyte signaling pathways that play a role in the pathogenesis of nephrotic syndrome (80). A better understanding of these pathways is needed to develop future targeted therapy. The high risk variant genotype of APOL1 gene (codes of apolipoprotein 1, known for its trypanolytic properties), was found to confer increased risk to kidney disease by gene-wide association studies (GWAS) (81–83). However, its association and role in nephrotic syndrome and podocyte biology are yet to be defined (80). New drugs on the horizon, such as losmapimod (p38 MAPK inhibitor), sparsentan (endothelin receptor type 1A antagonist), and biologics, such as adalimumab (anti-TNF-α) and abatacept (anti-CD80), hold promise in the treatment of steroid-resistant nephrotic syndrome and prevention of renal progression (79).

In summary, the seminal contributions of the ISKDC identified the importance of global collaborations in order to conduct meaningful multicenter trials aimed at effectively treating children with nephrotic syndrome, preventing renal progression and facilitating the opportunity for each child to reach their full potential and quality of life. The availability of advanced genetic and molecular applications to personalized medicine offer unique opportunities along this promising journey.

## Author Contributions

The authors contributed to the manuscript. Dr. AP is the first author as he did most of the work in reviewing the literature and formatting the style and content of the submission. Dr. AP is the corresponding author.

## Conflict of Interest Statement

The authors declare that the research was conducted in the absence of any commercial or financial relationships that could be construed as a potential conflict of interest.
